# Axial stent strut angle influences wall shear stress after stent implantation: analysis using 3D computational fluid dynamics models of stent foreshortening

**DOI:** 10.1186/1475-925X-4-59

**Published:** 2005-10-26

**Authors:** John F LaDisa, Lars E Olson, Douglas A Hettrick, David C Warltier, Judy R Kersten, Paul S Pagel

**Affiliations:** 1 Department of Pediatrics (Division of Cardiology), Stanford University, Palo Alto, California, USA; 2 Department of Anesthesiology, the Medical College of Wisconsin and the Clement J. Zablocki Veterans Affairs Medical Center, Milwaukee, Wisconsin, USA; 3 Department of Medicine (Division of Cardiovascular Diseases), the Medical College of Wisconsin and the Clement J. Zablocki Veterans Affairs Medical Center, Milwaukee, Wisconsin, USA; 4 Department of Pharmacology and Toxicology, the Medical College of Wisconsin and the Clement J. Zablocki Veterans Affairs Medical Center, Milwaukee, Wisconsin, USA; 5 Department of Biomedical Engineering, Marquette University, Milwaukee, Wisconsin, USA

**Keywords:** CFD, restenosis, neointimal hyperplasia, atherosclerosis, stent geometry

## Abstract

**Introduction:**

The success of vascular stents in the restoration of blood flow is limited by restenosis. Recent data generated from computational fluid dynamics (CFD) models suggest that the vascular geometry created by an implanted stent causes local alterations in wall shear stress (WSS) that are associated with neointimal hyperplasia (NH). Foreshortening is a potential limitation of stent design that may affect stent performance and the rate of restenosis. The angle created between axially aligned stent struts and the principal direction of blood flow varies with the degree to which the stent foreshortens after implantation.

**Methods:**

In the current investigation, we tested the hypothesis that stent foreshortening adversely influences the distribution of WSS and WSS gradients using time-dependent 3D CFD simulations of normal arteries based on canine coronary artery measurements of diameter and blood flow. WSS and WSS gradients were calculated using conventional techniques in ideal (16 mm) and progressively foreshortened (14 and 12 mm) stented computational vessels.

**Results:**

Stent foreshortening increased the intrastrut area of the luminal surface exposed to low WSS and elevated spatial WSS gradients. Progressive degrees of stent foreshortening were also associated with strut misalignment relative to the direction of blood flow as indicated by analysis of near-wall velocity vectors.

**Conclusion:**

The current results suggest that foreshortening may predispose the stented vessel to a higher risk of neointimal hyperplasia.

## Introduction

Stents are commonly used to treat coronary and peripheral vascular stenoses, but restenosis after implantation remains a persistent problem. The mechanisms responsible for restenosis have yet to be clearly elucidated[1,7,14]. Many stent designs are currently used in the clinical setting, and each of these designs has unique geometric and mechanical properties[5,13,36]. A correlation between stent geometry and the severity of subsequent neointimal hyperplasia has been described in animal models and humans[14,35,37,44]. We recently demonstrated that the number, width and thickness of stent struts and the deployment diameter and scaffolding created by an implanted stent substantially affects the area of the stented zone subjected to low wall shear stress (WSS) using time-dependent 3D computational fluid dynamics modeling[23,24]. We have also recently shown that these areas of low WSS predict subsequent development of neointimal hyperplasia *in vivo*[25].

Foreshortening (the difference between the desired and actual stent length after deployment) is a potential limitation of stent design that may affect stent performance and the rate of restenosis. The deployment of all types of stents is associated with deformation of struts about specific vertices within each stent mesh. Foreshortening varies with stent morphology because of differential deformation of interconnected struts during deployment[2,40]. The angle created between axially aligned stent struts and the principal direction of blood flow varies with the degree to which the stent foreshortens after implantation. Thus, theoretically deleterious variations in WSS distribution may occur in stent designs that demonstrate substantial degrees of foreshortening. A previous study demonstrated that strut angle influences cellular adhesion *in vitro*[12]. These data suggest that changes in strut angle resulting from foreshortening contribute to an altered flow environment that may be conducive to restenosis. In the current investigation, we tested the hypothesis that foreshortening adversely influences the distribution of WSS and WSS gradients previously implicated in neointimal hyperplasia using 3D computational fluid dynamics models.

## Methods

### Construction of stented computational vessels

**Figure 1:**
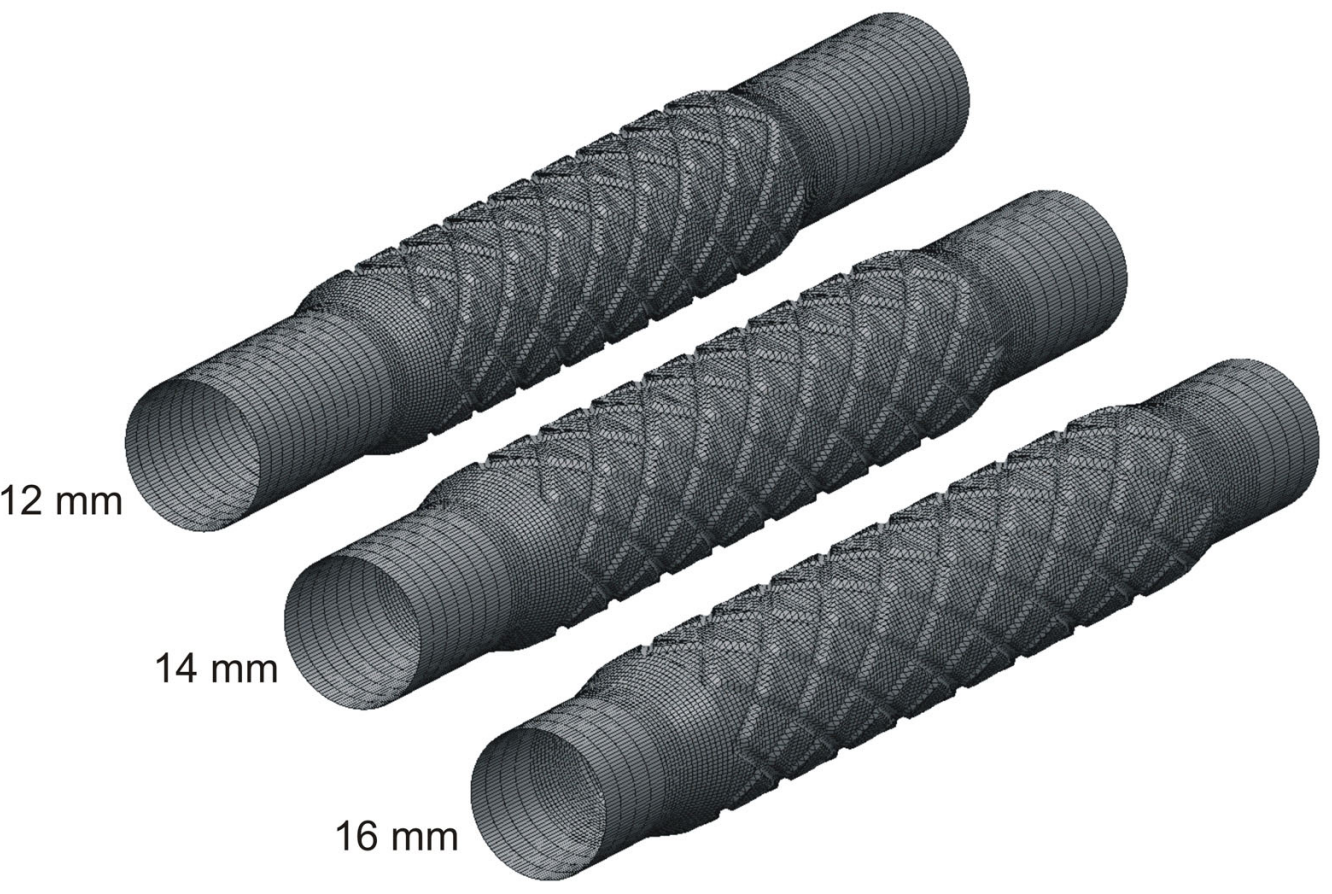
Computational vessels implanted with 12, 14 or 16 mm stents consisting of 8 axial and circumferential repeating strut sections that were deployed using a stent-to-artery diameter convention of 1.2 to 1. The diameter of the native vessel for all simulations was 2.74 mm. The computational vessel implanted with the 16 mm stent was designated as the ideal stent length after implantation and the 14 and 12 mm stents represent progressive degrees of foreshortening.

**Figure 2:**
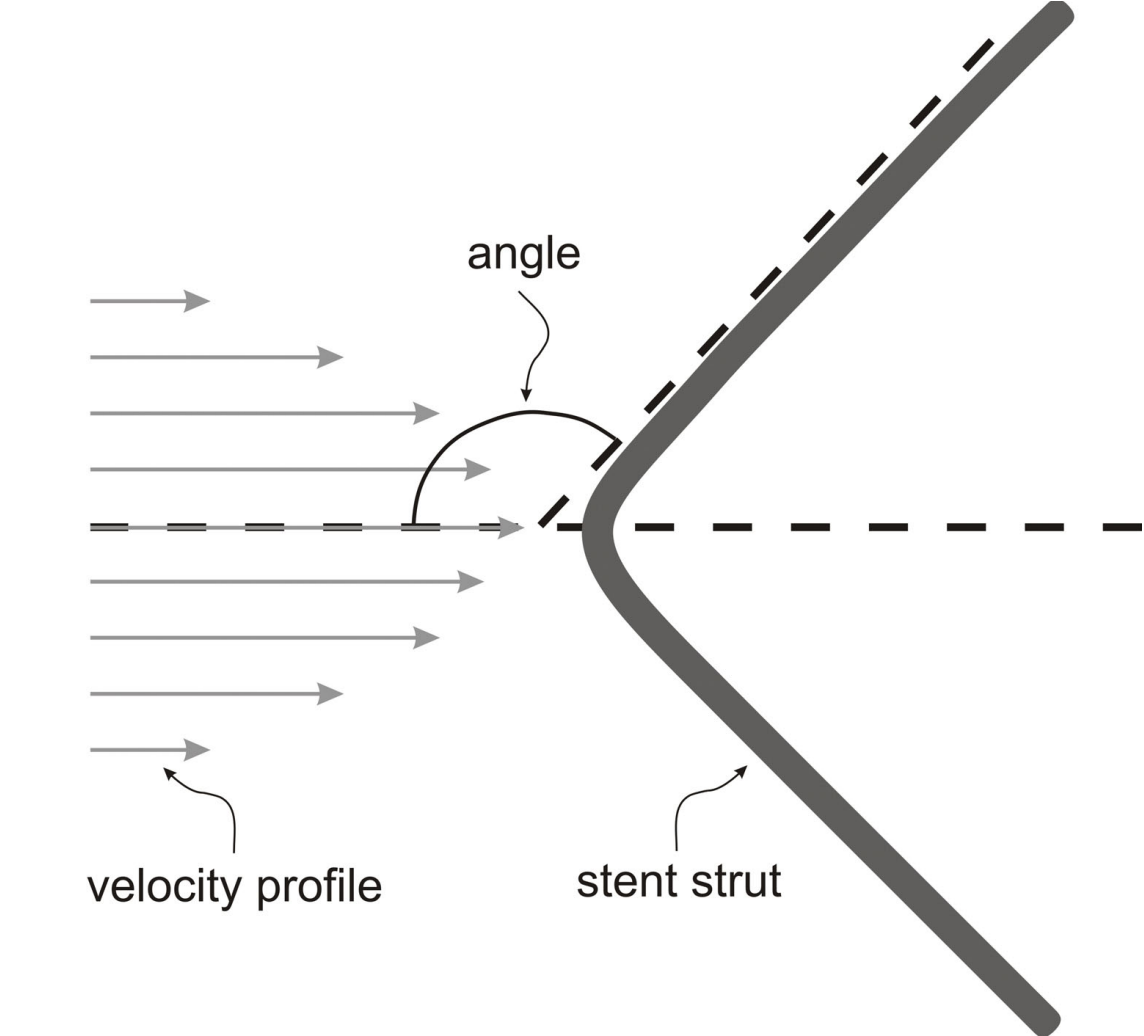
Schematic illustration demonstrating measurement of the stent strut angle with respect to the primary direction of blood flow.

A custom automated geometric construction and mesh generation algorithm was used to construct idealized computational arteries containing a slotted-tube stent embedded within a normal unstented computational vessel based on blood flow and diameter measurements obtained from canine left anterior descending coronary arteries[21]. The algorithm allows for the alteration of several geometric parameters including the stent length and diameter relative to that of the unstented portions of the computational vessel. The thickness and width of all stent struts were 0.096 and 0.197 mm, respectively. All computational vessels were composed of structured hexahedral control volumes arranged in a three-domain butterfly design that exploited symmetric stent and vessel properties to model one-fourth of the computational vessel. Three time-dependent simulations were performed in total. Computational vessels were created consisting of 8 axial and circumferential repeating strut sections that were deployed using a stent-to-artery diameter ratio of 1.2 to 1 (Figure 1). The diameter of the unstented portions of the computational vessels for all simulations was 2.74 mm. The computational vessel within the stented region conformed to the geometry of the implanted stent as previously documented[3,7,11,31]. The length of the computational stents were 12,14 or 16 mm where 16 mm represents the ideal stent length after implantation and the 14 and 12 mm stents represent progressive degrees of foreshortening, respectively. The amount of foreshortening used in the current models (12 to 25%) is consistent with stent foreshortening observed clinically[6,16]. The stent strut angles with respect to the primary direction of blood flow were 151°, 146° and 141°, for the 16, 14 and 12 mm stents, respectively. Maintaining the same number of axial and circumferential repeating strut units throughout each simulation facilitated the study of flow disturbances produced by this stent strut orientation angle with respect to the primary direction of blood flow (Figure 2).

### Computational model simulations

**Figure 3:**
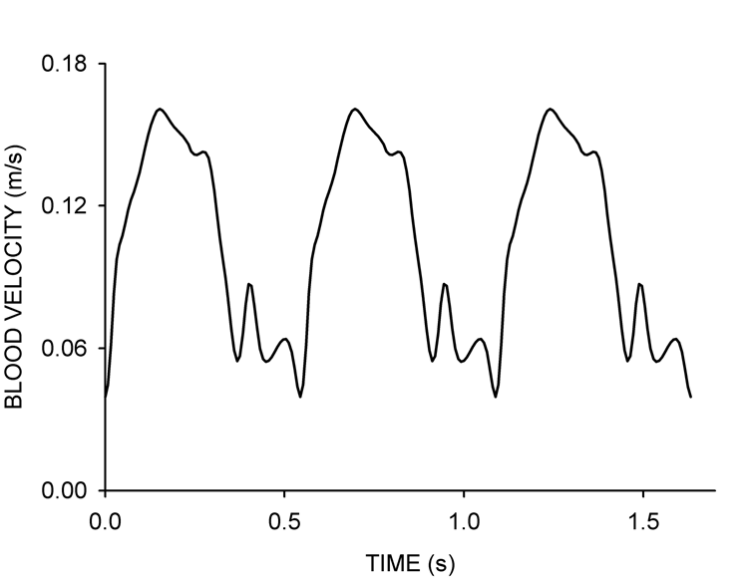
Blood flow velocity waveform measured in the proximal portion of a canine left anterior descending coronary artery and used for the time-dependent simulations conducted in the current investigation.

Simulations were performed using the commercially available software package CFD-ACE (CRDRC; Huntsville, AL, ). This software uses a finite volume approach to solve the Navier-Stokes equations at the center of each hexahedral control volume. Theoretical arteries were subjected to a blood flow velocity waveform obtained from a canine coronary artery under normal resting conditions (Figure 3). A plug flow velocity profile was imposed at the inlet of each vessel and additional length was added to all arteries to allow for fully developed flow[10,23]. An initial axial velocity of 0.105 m/s was imposed within the fluid domain at the start of each simulation to avoid a "cold start" and increase the likelihood of convergence. A zero pressure boundary condition was imposed at the outlet of the computational vessels. Simulations were conducted using a backward Euler temporal differencing method to investigate time-dependent changes in indices of WSS within the stented portion of each vessel. Computational simulations were conducted assuming a Newtonian, incompressible fluid with a density of 1.06 g/cm^3 ^and viscosity of 3.7 cP. The Reynolds and Womersley numbers for all of the simulations were approximately 105 and 2.91, respectively.

### Determination of wall shear stress indices

Wall shear stress was determined as the product of viscosity and shear rate. A detailed discussion of this calculation is presented elsewhere[24]. Briefly, the CFD-ACE flow solver calculates shear rate during incompressible flow using the second invariant of the strain rate tensor. Thus, shear rate () was determined as  where *u*, *v*, and *w *are the *x*, *y *and *z *components of velocity vector, **u**, respectively.

This definition accounts for pure shear as well as extensional or elongational deformation in the flow domain.

Spatial wall shear stress gradients (WSSG) were calculated during post-processing as discussed previously[15,23]. WSSG was used to quantify the influence of non-uniform hemodynamic forces on adjacent intravascular cells. Previous studies have suggested that this spatial WSS inhomogeneity may correlate with the location of neointimal hyperplasia *in vivo*[4,38,39] as was recently observed following chronic stent implantation into rabbit iliac arteries[25]. Frictional forces that act predominantly in axial and circumferential directions were most likely to cause expansion of intracellular gaps and disrupt intracellular junctions[28]. As a result, WSSG was calculated as , where τ_*w *_is the WSS in the axial (*z*) and circumferential (θ) directions, respectively. WSSG observed overlying stent struts were excluded from the current analysis because these areas do not acutely contain biologically active tissue immediately after acute stent implantation.

### Quantification of simulation results

The threshold for comparing distributions of low WSS between simulations was established at 5 dynes/cm^2 ^for comparison to previous work[23,24] and because vascular regions subjected to WSS below this value have been shown to strongly correlate with sites of intimal thickening[17,18]. Regions of low WSS were also expressed as percentages of the stent area within intrastrut regions in the proximal, middle and distal portions of the stent in order to determine perturbations produced by foreshortening and stent strut orientation. Near-wall velocity vectors were also visualized at spatial locations in the proximal, middle and distal portions of the stent to observe the behavior of blood flow in these regions.

WSSG have also been used previously to examine the hypothesis that normally confluent cells react to nonuniform distributions of WSS in a way that promotes neointimal hyperplasia[9,32,38]. The percentage of the vessel wall subjected to WSSG values above 20 dynes/cm^3 ^was quantified and compared between simulations in the current investigation. WSSG of this order of magnitude previously correlated with areas of neointimal hyperplasia in the toe region of an end-to-side arterial anastomosis[19,27,32].

## Results

### Mesh and time-step independence

Multiple simulations were performed to investigate spatial mesh independence. The spatial mesh density was four times greater in stented as compared to unstented regions of the computational vessels resulting in approximately 250,000 nodes per quarter of each computational artery. Results were considered to be spatially independent of the computational mesh when differences in the distributions of WSS were less than 6% between successive mesh densities[20,30]. Simulations were performed on a Dell Optiplex GX270 2.4 GHz workstation with 2 Gbyte of RAM that enabled convergence of simulations at a rate of approximately 25 time-steps per day. Time-step independence was examined by subjecting computational vessels to the coronary artery blood flow velocity waveform illustrated in Figure 3 using time-step increments of 10.9, 8.0 or 5.4 ms. Three consecutive cardiac cycles were allowed to reach simulation convergence, and distributions of WSS were compared at equivalent points during each cardiac cycle and between waveform permutations. The results demonstrated that a single cardiac cycle was sufficient to allow for the evolution of initial conditions, and simulation results became periodic shortly after the first cardiac cycle. A time step increment of 8.0 ms was sufficient to resolve temporal distributions of WSS within the stented and unstented regions of each vessel.

### Simulation results

Indices of WSS corresponding to the mean blood flow velocity during the cardiac cycle are shown in Table 1. Lower WSS was observed within the stented region of all simulations, and stagnation zones occurred around stent struts. The percentage of intrastrut area exposed to distributions of WSS < 5 dynes/cm^2 ^was greatest in the proximal portion of each stent independent of length and strut orientation (Table 1). These intrastrut areas of low WSS were least pronounced in the distal portion of the stents. The percentage of intrastrut area exposed to WSS < 5 dynes/cm^2 ^within a particular intrastrut region decreased as the length of the stent increased. Conversely, the total area of the stent exposed to WSS < 5 dynes/cm^2 ^increased as the stent length increased. However, when normalized by the length of the stent, the percentage of the computational vessel subjected to WSS < 5 dynes/cm^2 ^modestly increased with the degree of foreshortening (0.78 as compared to 0.71 mm^2 ^for 16 and 12 mm stents, respectively).

**Table 1:** Stent properties and indices of wall shear stress

**Stent Length (mm)**	**16**	**14**	**12**
**Luminal surface area covered by the stent (%)**	24	22	19
**Stent strut angle with respect to primary flow direction (°)**	151	146	141
**Proximal Intrastrut WSS < 5 dynes/cm^2 ^(%)**	48	52	55
**Middle Intrastrut WSS < 5 dynes/cm^2 ^(%)**	18	46	50
**Distal Intrastrut WSS < 5 dynes/cm^2 ^(%)**	12	21	48
**Total area exposed to WSS < 5 dynes/cm^2 ^(mm^2^)**	117	111	96.8
**Total area exposed to WSSG > 20 dynes/cm^3 ^(mm^2^)**	89.3	72.5	67.4
**Normalized area exposed to WSS < 5 dynes/cm^2^**	0.71	0.77	0.78
**Normalized area exposed to WSSG > 20 dynes/cm^3^**	0.54	0.50	0.54
**WSSG_max _(dynes/cm^3^)**	248	293	395

Abbreviations: WSS = wall shear stress; WSSG = wall shear stress gradient

The total area of the computational vessel subjected to WSSG greater than 20 dynes/cm^3 ^increased as the stent length increased (67.4 versus 89.3 dynes/cm^3 ^for 12 and 16 mm stents, respectively). In addition, the maximum WSSG observed throughout the stented region decreased as stent length increased (395, 293 and 248 dynes/cm^3 ^for the 12, 14 and 16 mm stents, respectively). When normalized by the length of the stent, the portion of vessels subjected to elevated WSSG was similar for each computational vessel simulation (Table 1).

**Figure 4:**
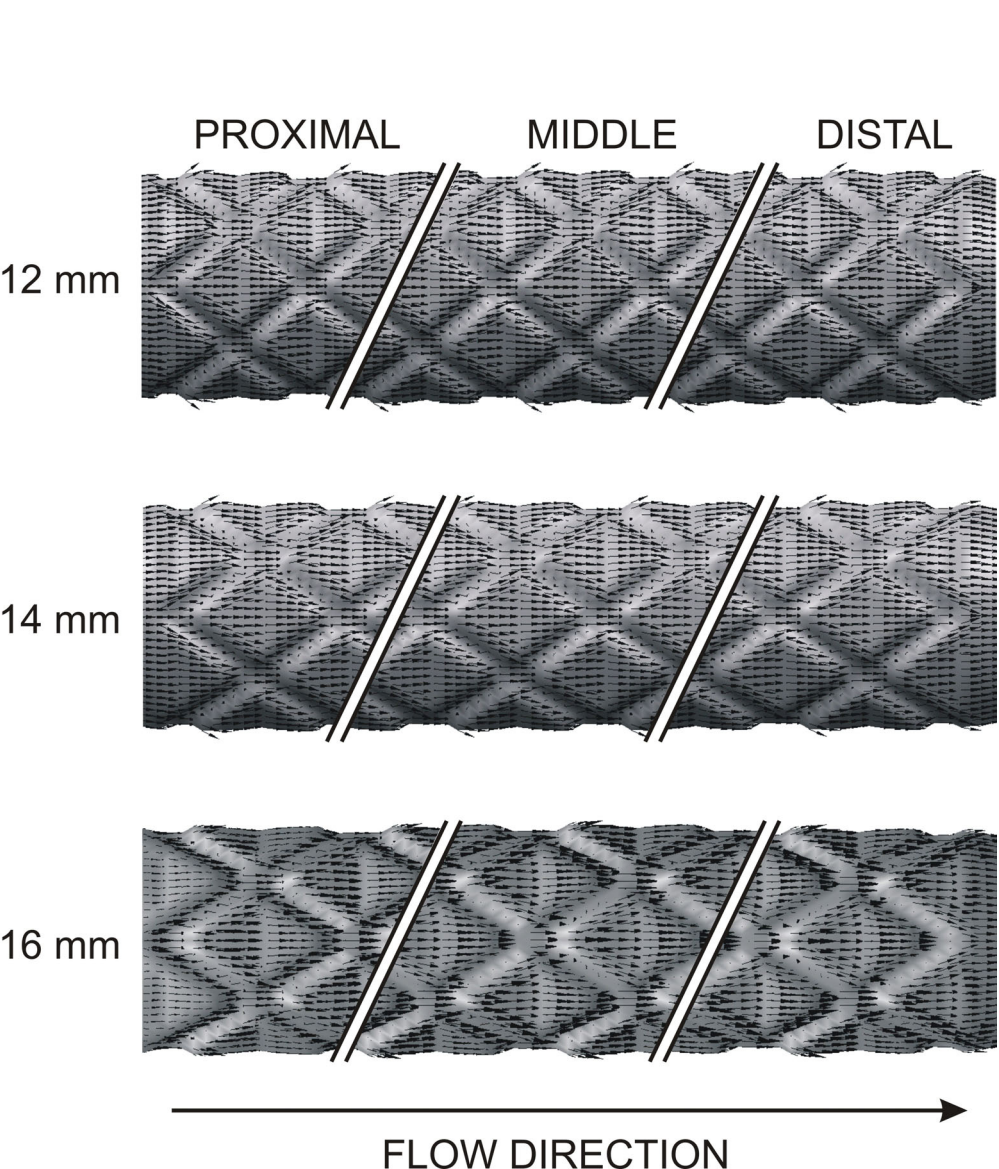
Value-weighted near-wall velocity vectors in the proximal, middle and distal portions of the stent resulting from the unique strut orientation angles corresponding to ideal (16 mm) and progressively foreshortened stents (14 and 12 mm).

Value-weighted near-wall velocity vectors in the first proximal, middle and last distal repeating stent unit resulting from the unique strut orientation of each of the stents are illustrated in Figure 4. Reductions in the size of the vectors in the proximal as compared to the middle region resulted from the increase in luminal diameter within the stented region and account for the pronounced low WSS found in the proximal portion of the computational stent in all simulations. Adjacent blood layers appear to converge before entering each repeating stent unit and diverge after entering each axial diamond. This divergence accounted for reductions in WSS adjacent to stent struts. Figure 4 also demonstrates that the 12 mm stent foreshortening simulation produced large deviations in value-weighted near-wall velocity vectors adjacent to stent struts, in contrast to the observations with computational stents deployed to an ideal length of 16 mm.

**Figure 5:**
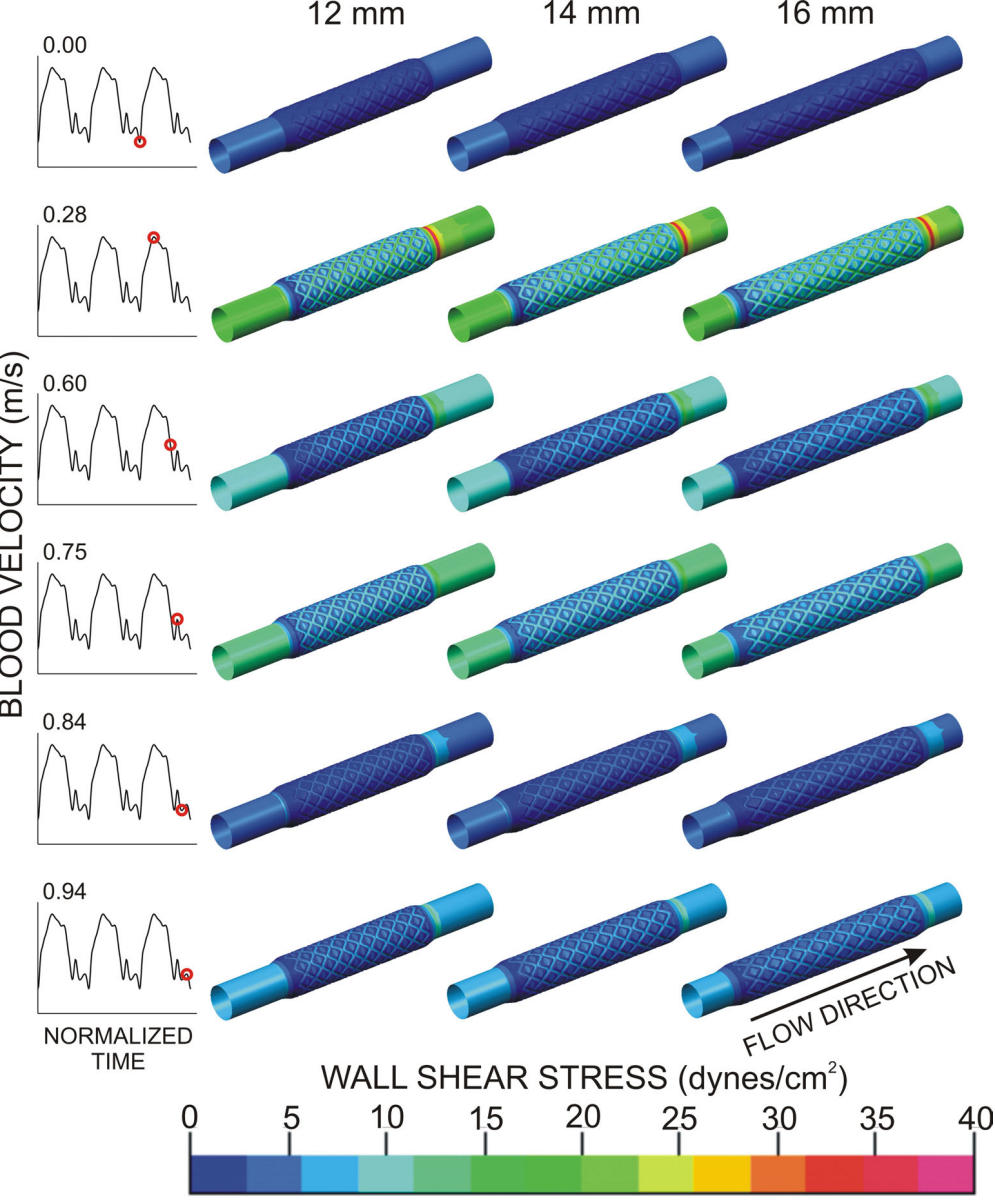
Time-dependent alterations in spatial wall shear stress throughout the cardiac cycle in ideal stents (16 mm) and those with progressive degrees of foreshortening (14 and 12 mm).

Time-dependent alterations in the spatial distributions of WSS throughout the cardiac cycle are illustrated in Figure 5. Stagnation regions observed adjacent to stent struts developed immediately before blood flow deceleration at the local and global maxima of the flow waveform (rows 2, 4 and 6). These stagnation regions were more pronounced in the 12 and 14 mm computational stents as compared to the 16 mm simulation.

## Discussion

Increasing evidence suggests that the geometry and physical properties of a deployed stent may influence the local blood flow environment and alter distributions of WSS after implantation, thereby rendering certain areas of the vessel wall more susceptible to neointimal hyperplasia and subsequent restenosis[11,29,31,43]. Our laboratory has previously demonstrated that the number, width and thickness of strut struts, the stent-to-artery deployment diameter, and stent scaffolding influence the area of a vessel exposed to low WSS and elevated spatial and temporal WSSG using computational fluid dynamics models generated from measured *in vivo *data[20,23,24]. These results suggested that stent geometry profoundly affects fluid dynamics that correlate with the spatial and temporal localization of neointimal hyperplasia as we recently reported using a combined approach of chronic iliac artery stent implantation *in vivo*, microfocal x-ray CT imaging, computational fluid dynamics and vascular histology[22,25,26].

The current results confirm and extend our previous observations and further demonstrate the importance of stent geometry on intravascular fluid dynamics. The current findings with 3D computational fluid dynamics modeling demonstrate that stent foreshortening increases the area of the luminal surface exposed to low WSS and elevated spatial WSSG. These data strongly suggest that stent foreshortening may precipitate more pronounced development of neointimal hyperplasia as compared to a stent deployed to its ideal length. This hypothesis remains to be tested *in vivo*, however. The angle created between axially aligned stent struts and the principal direction of blood flow varied with the deployed stent length. The current results using computational foreshortened stents and those deployed to their ideal length demonstrate that struts of the foreshortened stents were progressively misaligned with the direction of blood flow. This strut misalignment within the flow domain also substantially influenced the behavior of near-wall velocity vectors and contributed to the observed increases in deleterious distributions of WSS and WSSG in foreshortened as compared to ideal computational stent architecture. Stent foreshortening effectively increases the number of struts per unit length of a vessel, and we have previously demonstrated that such an increase in strut number is associated with greater exposure of the luminal surface to low WSS and elevated WSSG[23]. These previous and current observations are consistent with the intuitive notion that optimal stent design should assure that the vast majority of struts are aligned more parallel to blood flow in order to minimize changes in laminar blood flow characteristics and WSS distributions.

Although the normalized luminal area exposed to WSS < 5 dynes/cm^2 ^was similar for each of the stents computationally modeled in the current investigation, it is important to consider which of the parameters reported in Table 1 are most likely to influence the local cellular response and be associated with neointimal hyperplasia. The table clearly indicates that the angle of stent struts with respect to the primary flow direction strongly influences the intrastrut area of the vessel wall exposed to low WSS. Cells in these pronounced areas of low WSS are more likely to participate in the neointimal response. Normalizing the total area of the vessel exposed to low WSS by the length provides a less sensitive indication of the local impact on cells within the stented region that are likely to contribute to neointimal hyperplasia.

The current results require interpretation within the constraints of several potential limitations. Stents were implanted in idealized computational representations of healthy vessels and the results would likely differ if the study was performed using computational models of vascular disease. From a clinical perspective, stent diameter typically increases with the degree of foreshortening. Such an increase in stent diameter would be expected to intrinsically produce a reduction in blood velocity, and hence WSS, within the stented region. We conducted the current investigation using a consistent diameter within the stented region of each computational vessel in order to isolate the influence of the incident strut angle on indices of WSS independent of changes in vessel diameter. As a result, the current results may actually underestimate the relative amount of the computational vessels subjected to low WSS and elevated spatial WSSG as a result of this observed clinical increase in diameter often associated with foreshortening. The current investigation was conducted using a rigid-wall approximation and a simplified outlet boundary condition. Previous studies have demonstrated that implantation of 16 mm slotted-tube stents into canine epicardial coronary arteries *in vivo *reduced vessel compliance to zero within the stented region[21] and that wall deformability does not greatly alter the velocity field under normal conditions[34]. Nevertheless, the results may differ from those observed clinically as a result of arterial compliance proximal and distal to the stented region and the ability of the distal vasculature to vasodilate in response to local metabolic need, thus altering the upstream resistance through the stented segment.

The potential clinical applicability of the current results in lieu of the widespread use of drug-eluting stents to treat vascular stenoses also deserves comment. Recent reviews suggested that drug-eluting stents may simply delay restenosis, but do not facilitate healing of the intima that is damaged by prior vascular disease or the stent implantation procedure. Thus, restenosis rates with drug-eluting stent may ultimately be similar to bare metal stents[8,33,41,42]. Moreover, this drug-eluting technology may not be applicable to all patient populations and locations within the arterial vasculature[8,33]. The current results further support the hypothesis that local flow patterns created by the stent should also be considered during stent design so as to minimize indices of fluid dynamics implicated in neointimal hyperplasia and to avoid the use of exogenous cytotoxic or antiproliferative agents.

## Conclusion

In summary, the current results using 3D computational fluid dynamics modeling indicate that foreshortening-induced orientation of stent struts with respect to the direction of blood flow is associated with increases in the area of the vessel subjected to low WSS and high WSSG, factors that have been previously correlated with subsequent neointimal hyperplasia *in vivo*. The change in incident strut angle with respect to the predominant flow direction was modest between modeled foreshortened as compared to ideal computational stents (141 to 151°), but increasing the degree of foreshortening reduced the axial alignment angle of stent struts and produced greater intrastrut areas of low WSS and elevated WSSG. Stent geometries that successfully restore distal perfusion but produce the least disruption to the native flow environment may ultimately provide maximum chronic vessel patency.

## Authors' contributions

JFL planned and conducted the experiments from which the blood flow and diameter measurements used in the current investigation were obtained, created the automated geometric construction and mesh generation algorithm, formulated fluid dynamics models, conducted simulations and wrote drafts of the manuscript. LEO assisted with the formulation of fluid dynamics models, planning and completion of simulations, and helped with data analysis. DAH assisted in the design of the computational investigation, formulated fluid dynamics models, and helped with data analysis. JRK assisted in data analysis and critical revisions of manuscript. DCW also assisted in data analysis and critical revisions of the manuscript. PSP critically reviewed the design of the computational study and experiments from which the measurements used in the current investigation were obtained, fluid dynamics theory, and analysis of results and also provided multiple critical revisions of several drafts of the manuscript, including the final submitted manuscript. All authors read and agreed to the submission of the manuscript in its current form.
